# Supplementary material for: Ontogenetic growth in the crania of Exaeretodon argentinus (Synapsida: Cynodontia) captures a dietary shift
Source: PeerJ. 2022 Oct 21;10:e14196. doi: 10.7717/peerj.14196 (PMC9590418; doi:10.7717/peerj.14196)
Supplement: Supplemental Information 1 [file peerj-10-14196-s001.docx]

SUPPLEMENT TO:

ONTOGENETIC GROWTH IN THE CRANIA OF *EXAERETODON ARGENTINUS* (SYNAPSIDA: CYNODONTIA) CAPTURES DIETARY SHIFTS

Brenen M. Wynd^1,*^, Fernando Abdala^2^, and Sterling J. Nesbitt^1^

^1^Virginia Tech, Blacksburg, Virginia, U.S.A.; bmwynd@vt.edu

^2^Universidad Nacional de Tucumán, Miguel Lillo 205, 4000, Tucumán, Argentina

**Additional allometric figures**

Here, we include the bivariate plots for all of the measurements for *Exaeretodon argentinus*. The parameter estimates are reported in the manuscript. We report the coefficient of allometry for each model (α_GLMM_ and α_OLS_) in the figure captions.


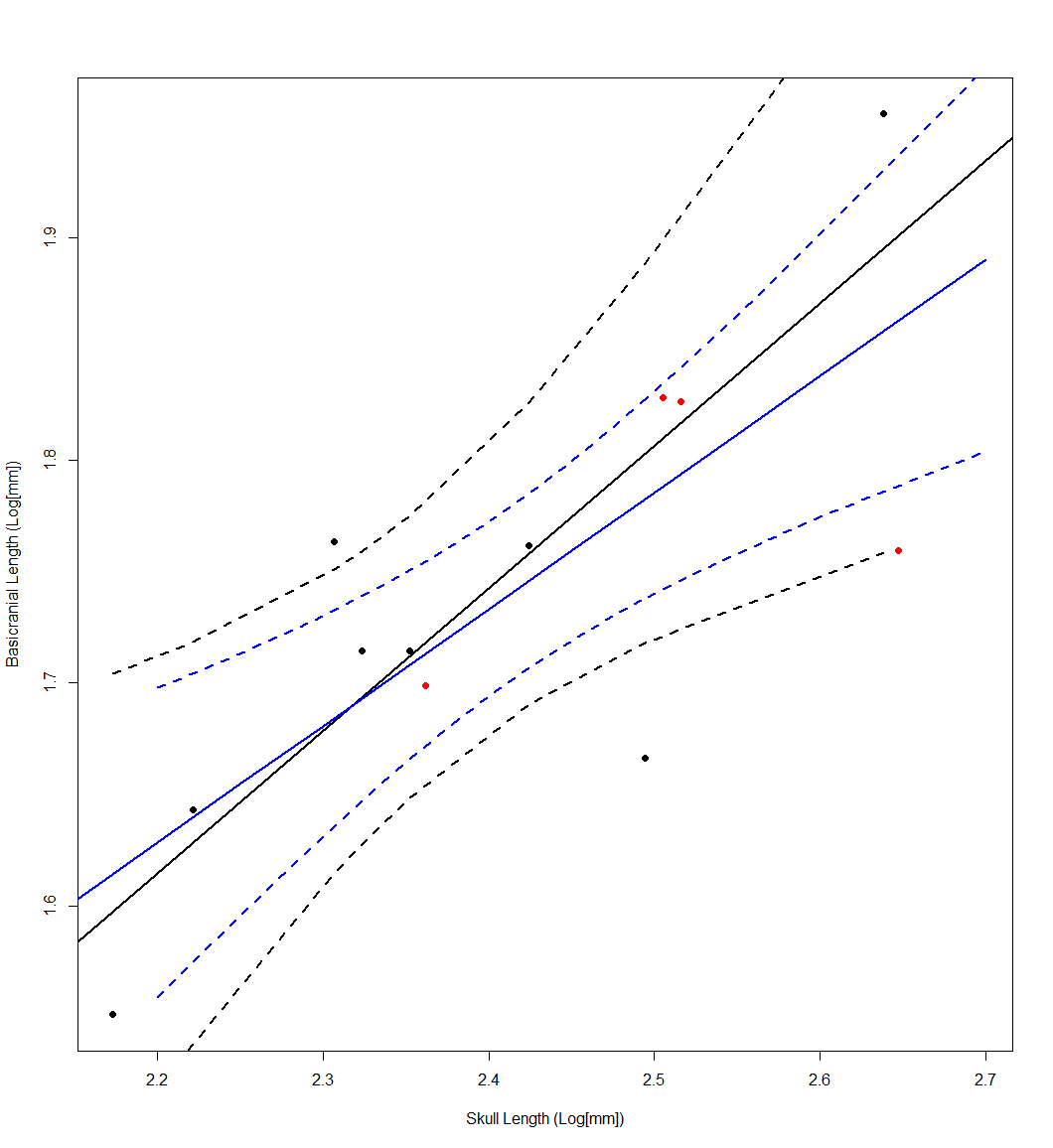


FIGURE S1: Relationship between basal skull length and basicranial length. Distorted measurements are represented as red dots, and undistorted measurements are represented as black dots. Ordinary least squares regression of only the black points is shown in black, and the GLMM of all samples is shown in blue. 95% confidence intervals are reported for both OLS and GLMM as dotted lines of corresponding color. α_GLMM_: 0.5238^**^; and α_OLS_: 0.6402^*^. Marginally significant models are marked via ^*^, and significance is marked via ^**^.


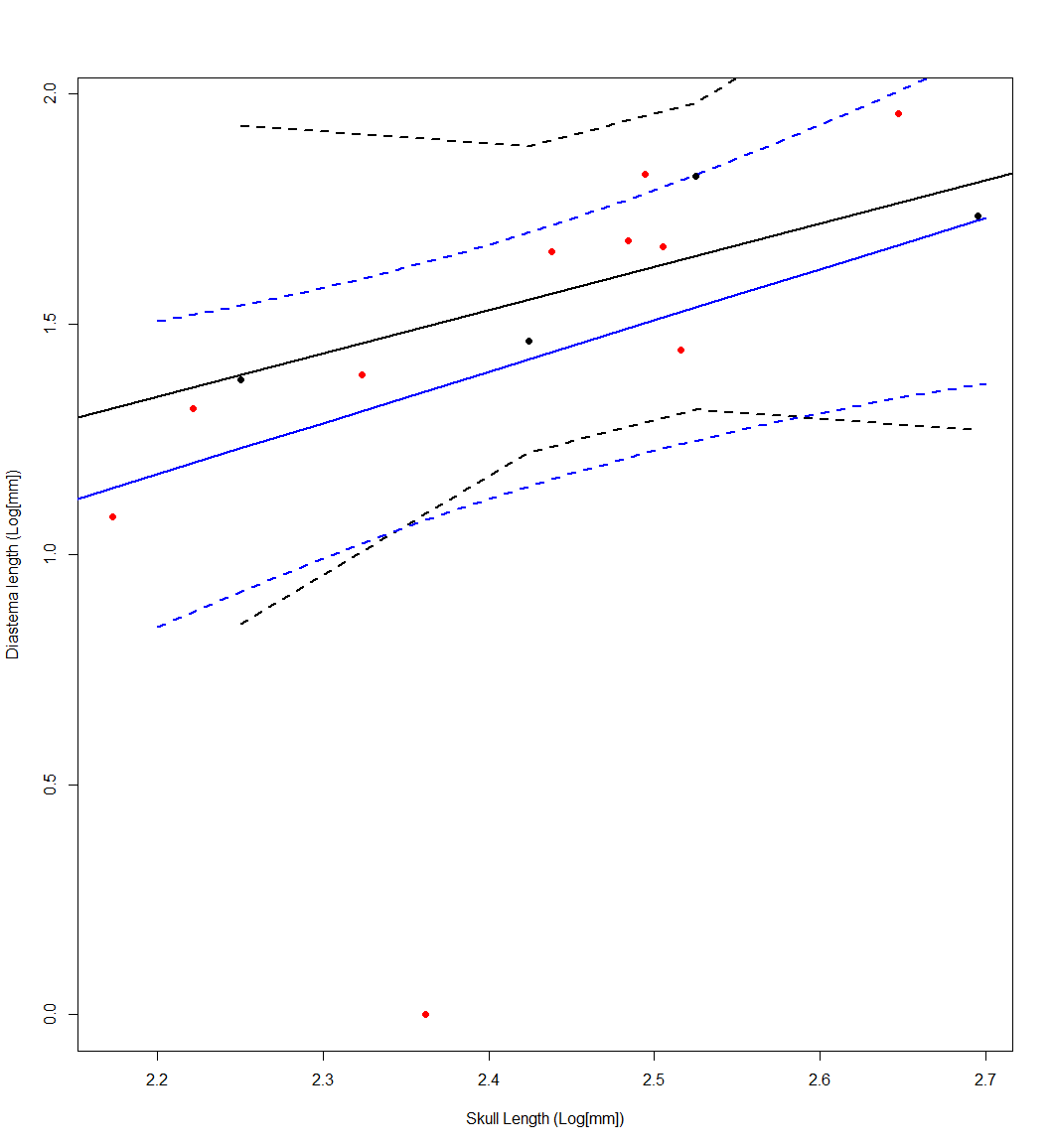


FIGURE S2: Relationship between basal skull length and diastema length. Distorted measurements are represented as red dots, and undistorted measurements are represented as black dots. Ordinary least squares regression of only the black points is shown in black, and the GLMM of all samples is shown in blue. 95% confidence intervals are reported for both OLS and GLMM as dotted lines of corresponding color. α_GLMM_: 1.1151; and α_OLS_: 0.9384. Marginally significant models are marked via ^*^, and significance is marked via ^**^.


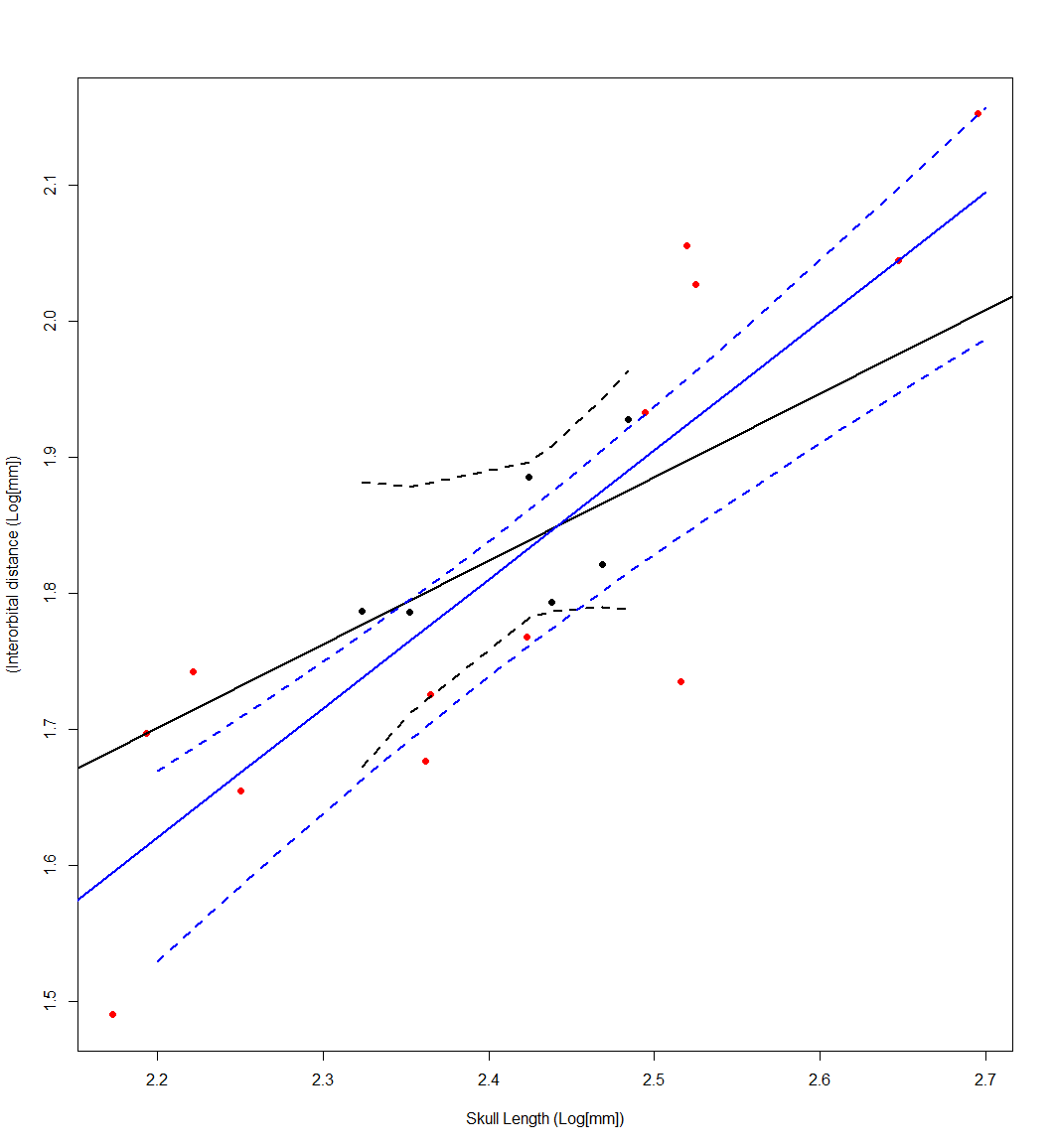


FIGURE S3: Relationship between basal skull length and interorbital distance. Distorted measurements are represented as red dots, and undistorted measurements are represented as black dots. Ordinary least squares regression of only the black points is shown in black, and the GLMM of all samples is shown in blue. 95% confidence intervals are reported for both OLS and GLMM as dotted lines of corresponding color. α_GLMM_: 0.9488; and α_OLS_: 0.6144. Marginally significant models are marked via ^*^, and significance is marked via ^**^.


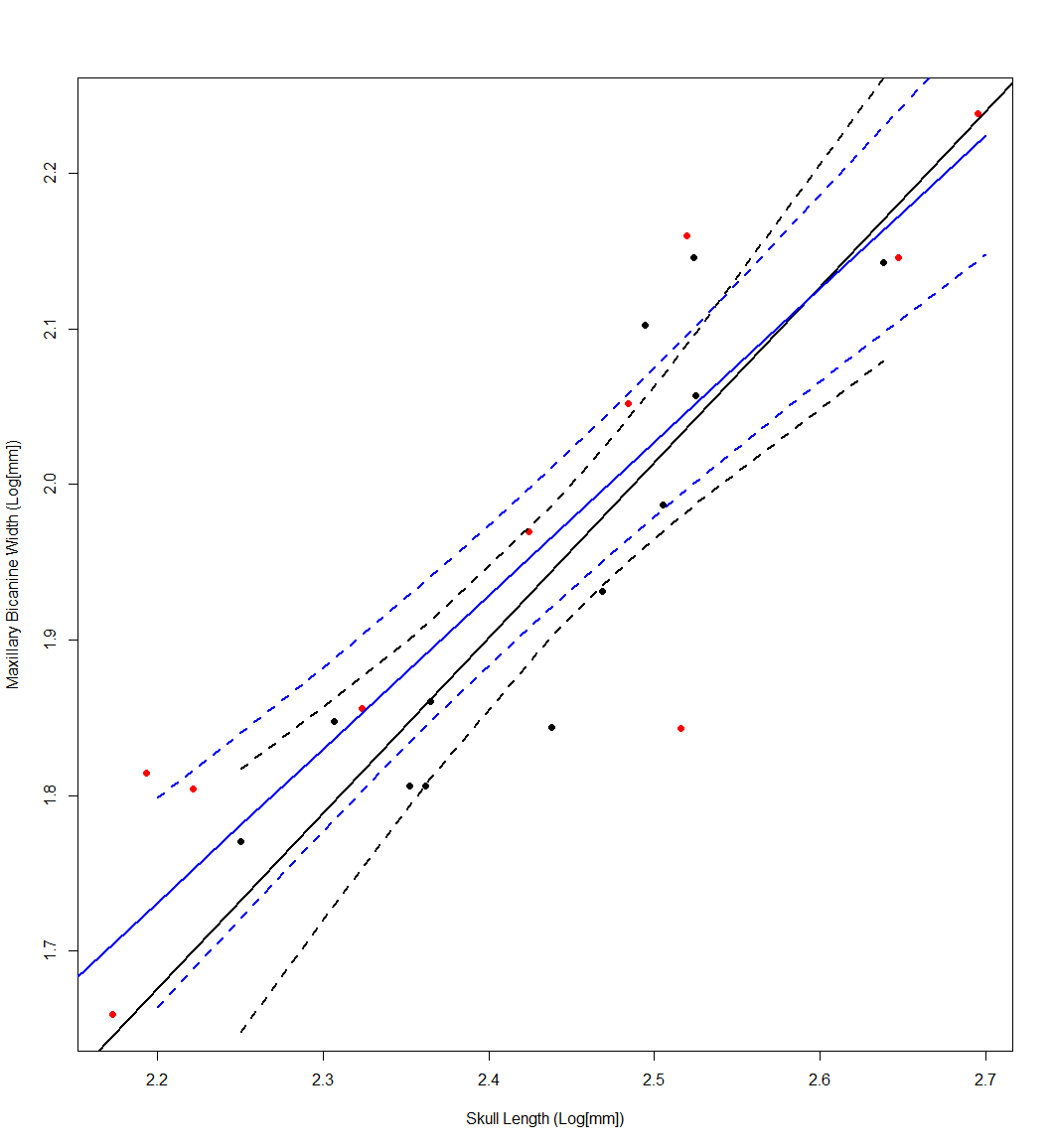


FIGURE S4: Relationship between basal skull length and maxillary bicanine width. Distorted measurements are represented as red dots, and undistorted measurements are represented as black dots. Ordinary least squares regression of only the black points is shown in black, and the GLMM of all samples is shown in blue. 95% confidence intervals are reported for both OLS and GLMM as dotted lines of corresponding color. α_GLMM_: 0.9877; and α_OLS_: 0.1.1296. Marginally significant models are marked via ^*^, and significance is marked via ^**^.


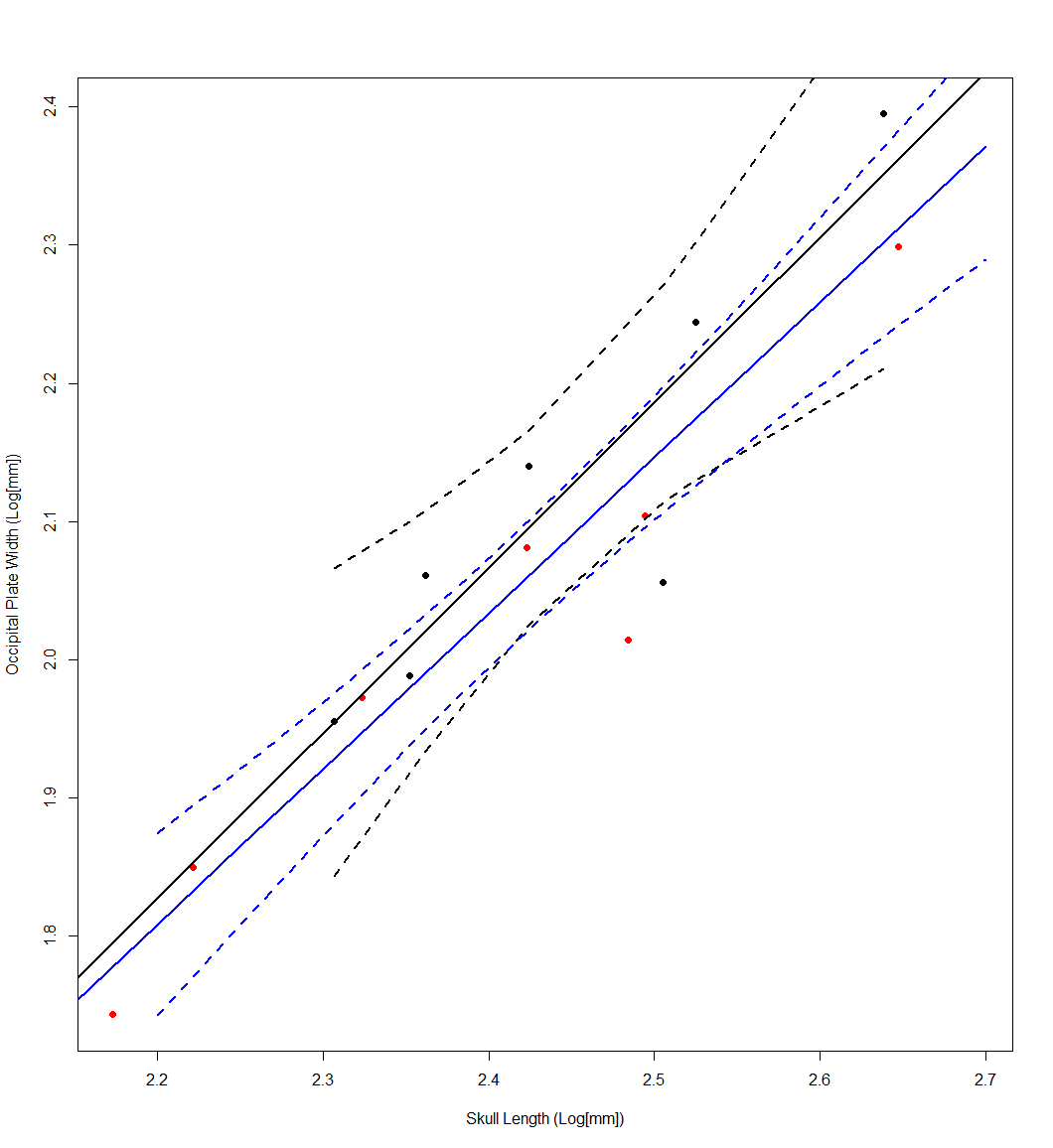


FIGURE S5: Relationship between basal skull length and occipital plate width. Distorted measurements are represented as red dots, and undistorted measurements are represented as black dots. Ordinary least squares regression of only the black points is shown in black, and the GLMM of all samples is shown in blue. 95% confidence intervals are reported for both OLS and GLMM as dotted lines of corresponding color. α_GLMM_: 1.1273; and α_OLS_: 1.1978. Marginally significant models are marked via ^*^, and significance is marked via ^**^.


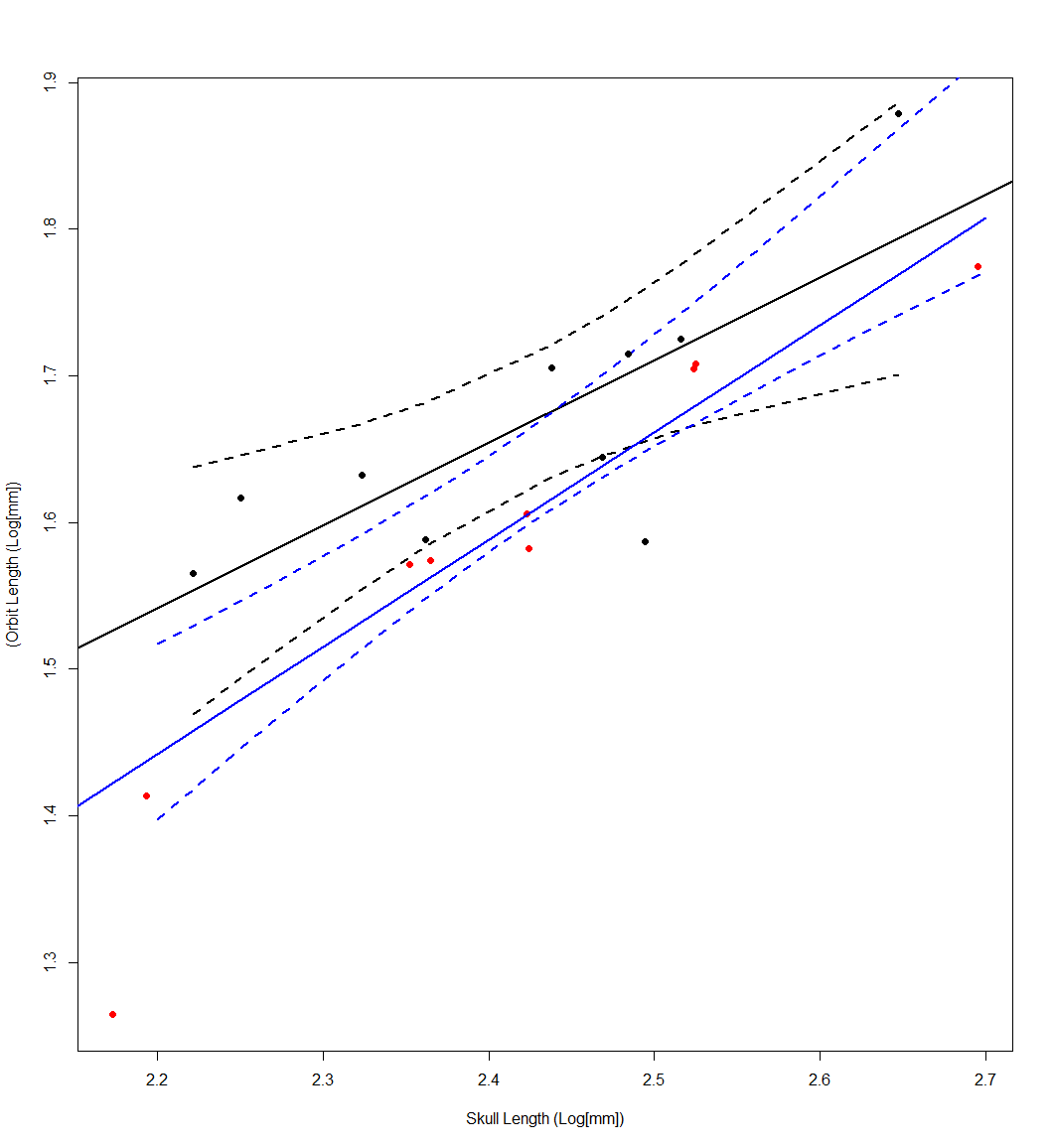


FIGURE S6: Relationship between basal skull length and orbit length. Distorted measurements are represented as red dots, and undistorted measurements are represented as black dots. Ordinary least squares regression of only the black points is shown in black, and the GLMM of all samples is shown in blue. 95% confidence intervals are reported for both OLS and GLMM as dotted lines of corresponding color. α_GLMM_: 0.7321^**^; and α_OLS_: 0.564^**^. Marginally significant models are marked via ^*^, and significance is marked via ^**^.


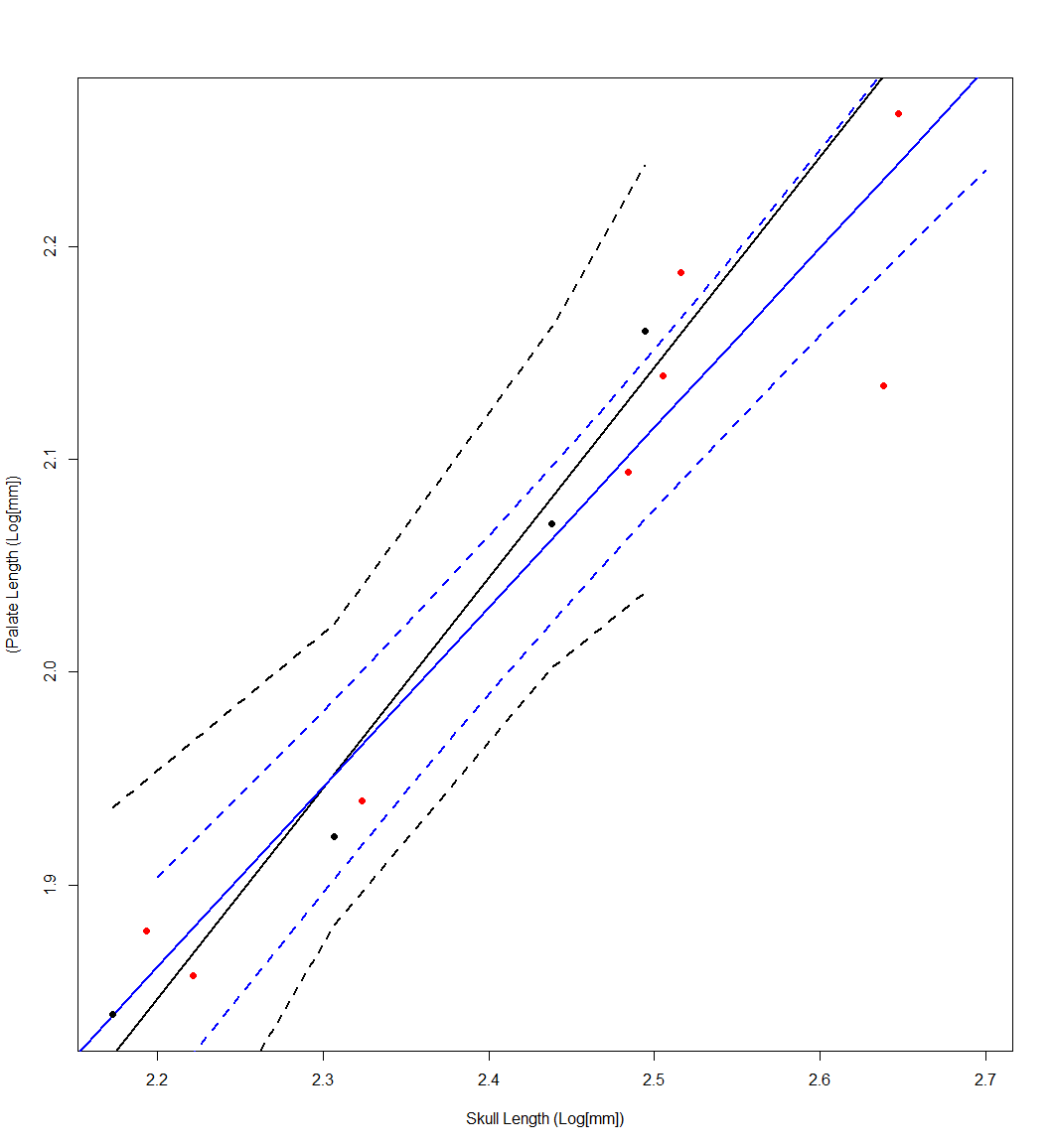


FIGURE S7: Relationship between basal skull length and palate length. Distorted measurements are represented as red dots, and undistorted measurements are represented as black dots. Ordinary least squares regression of only the black points is shown in black, and the GLMM of all samples is shown in blue. 95% confidence intervals are reported for both OLS and GLMM as dotted lines of corresponding color. α_GLMM_: 0.8439^*^; and α_OLS_: 0.9889. Marginally significant models are marked via ^*^, and significance is marked via ^**^.


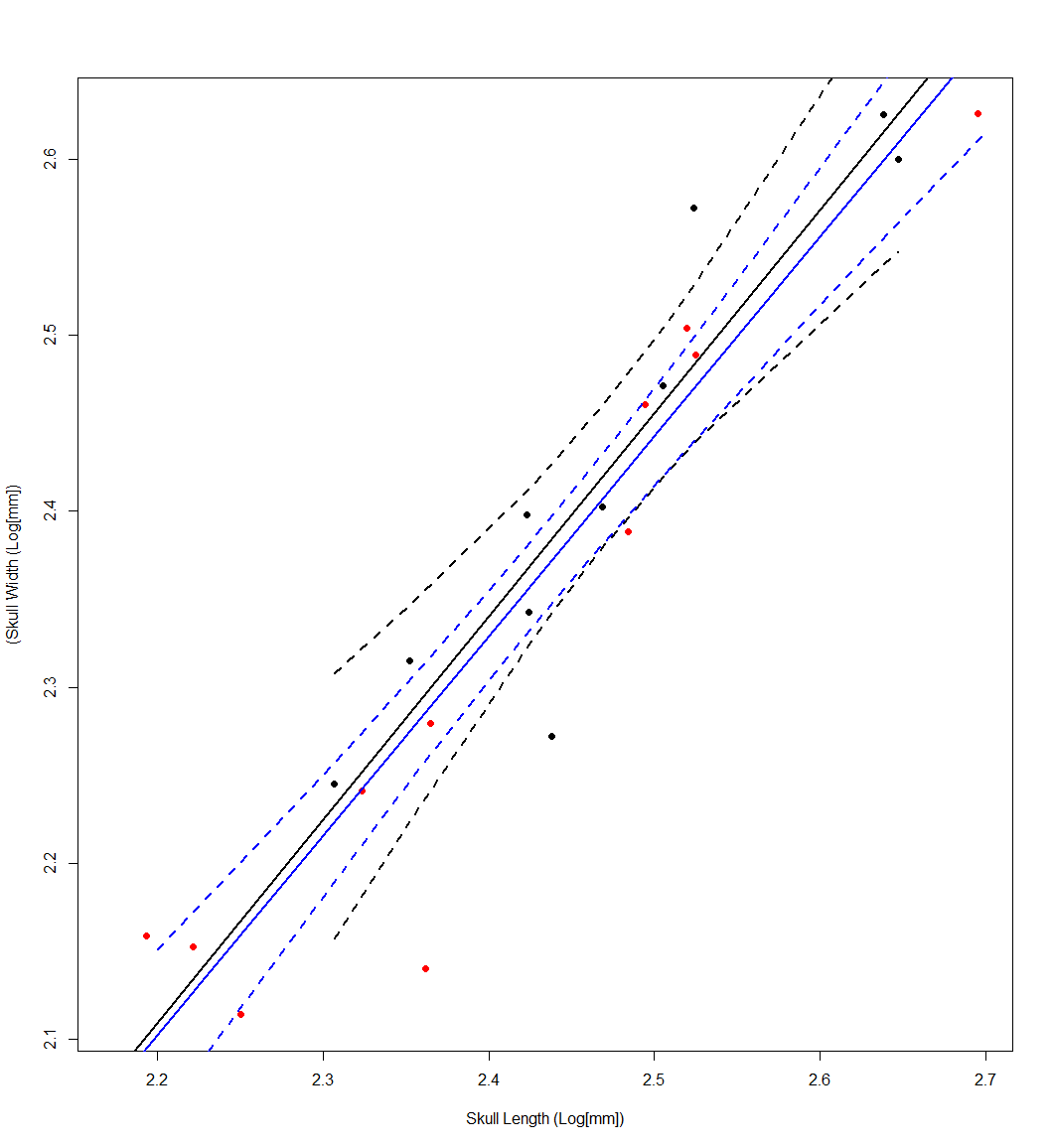


FIGURE S8: Relationship between basal skull length and skull width. Distorted measurements are represented as red dots, and undistorted measurements are represented as black dots. Ordinary least squares regression of only the black points is shown in black, and the GLMM of all samples is shown in blue. 95% confidence intervals are reported for both OLS and GLMM as dotted lines of corresponding color. α_GLMM_: 1.1343; and α_OLS_: 1.1553^*^. Marginally significant models are marked via ^*^, and significance is marked via ^**^.


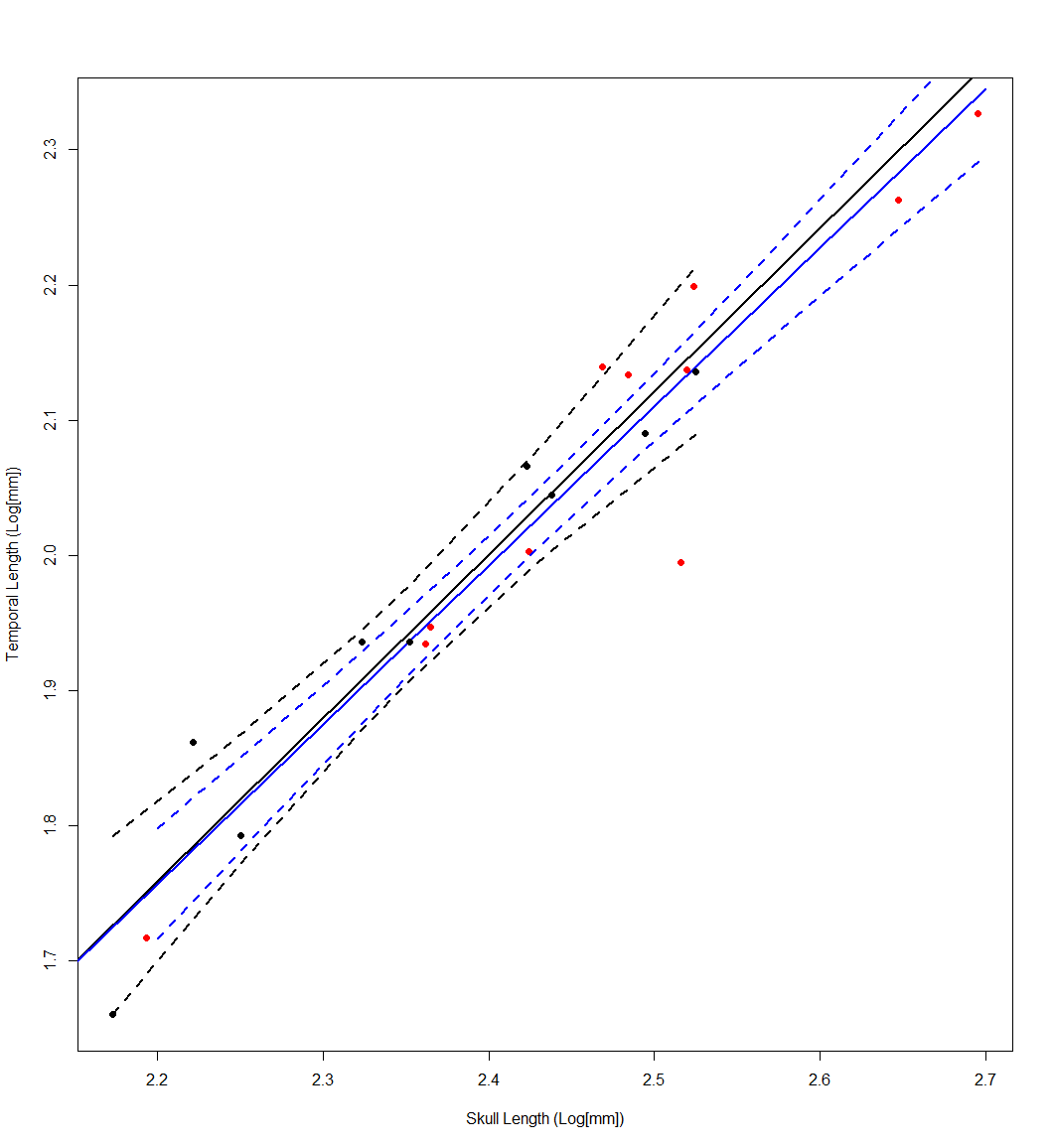


FIGURE S9: Relationship between basal skull length and temporal length. Distorted measurements are represented as red dots, and undistorted measurements are represented as black dots. Ordinary least squares regression of only the black points is shown in black, and the GLMM of all samples is shown in blue. 95% confidence intervals are reported for both OLS and GLMM as dotted lines of corresponding color. α_GLMM_: 1.1761^**^; and α_OLS_: 1.2077. Marginally significant models are marked via ^*^, and significance is marked via ^**^.


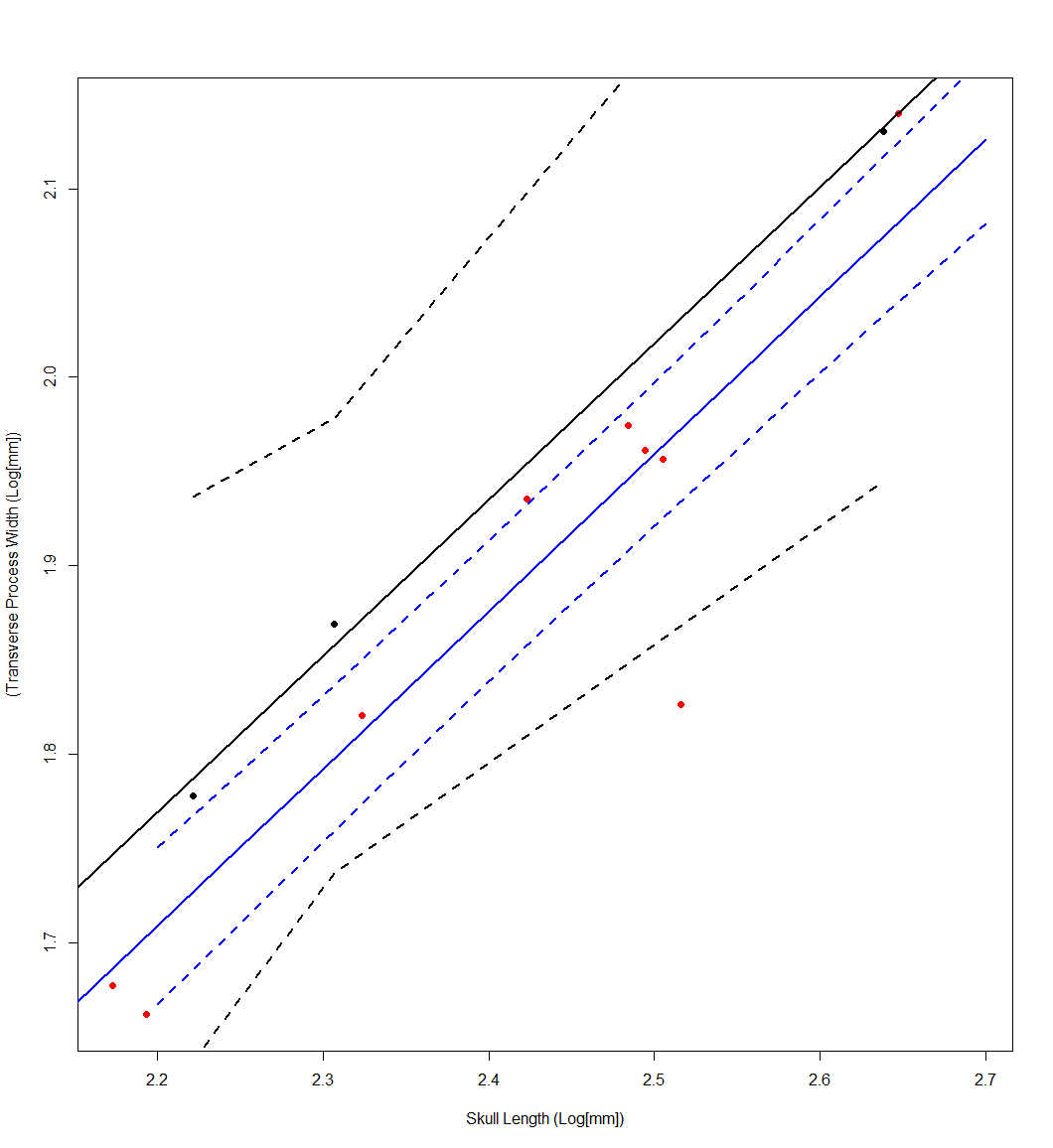


FIGURE S10: Relationship between basal skull length and transverse process width. Distorted measurements are represented as red dots, and undistorted measurements are represented as black dots. Ordinary least squares regression of only the black points is shown in black, and the GLMM of all samples is shown in blue. 95% confidence intervals are reported for both OLS and GLMM as dotted lines of corresponding color. α_GLMM_: 0.83507; and α_OLS_: 0.82948. Marginally significant models are marked via ^*^, and significance is marked via ^**^.


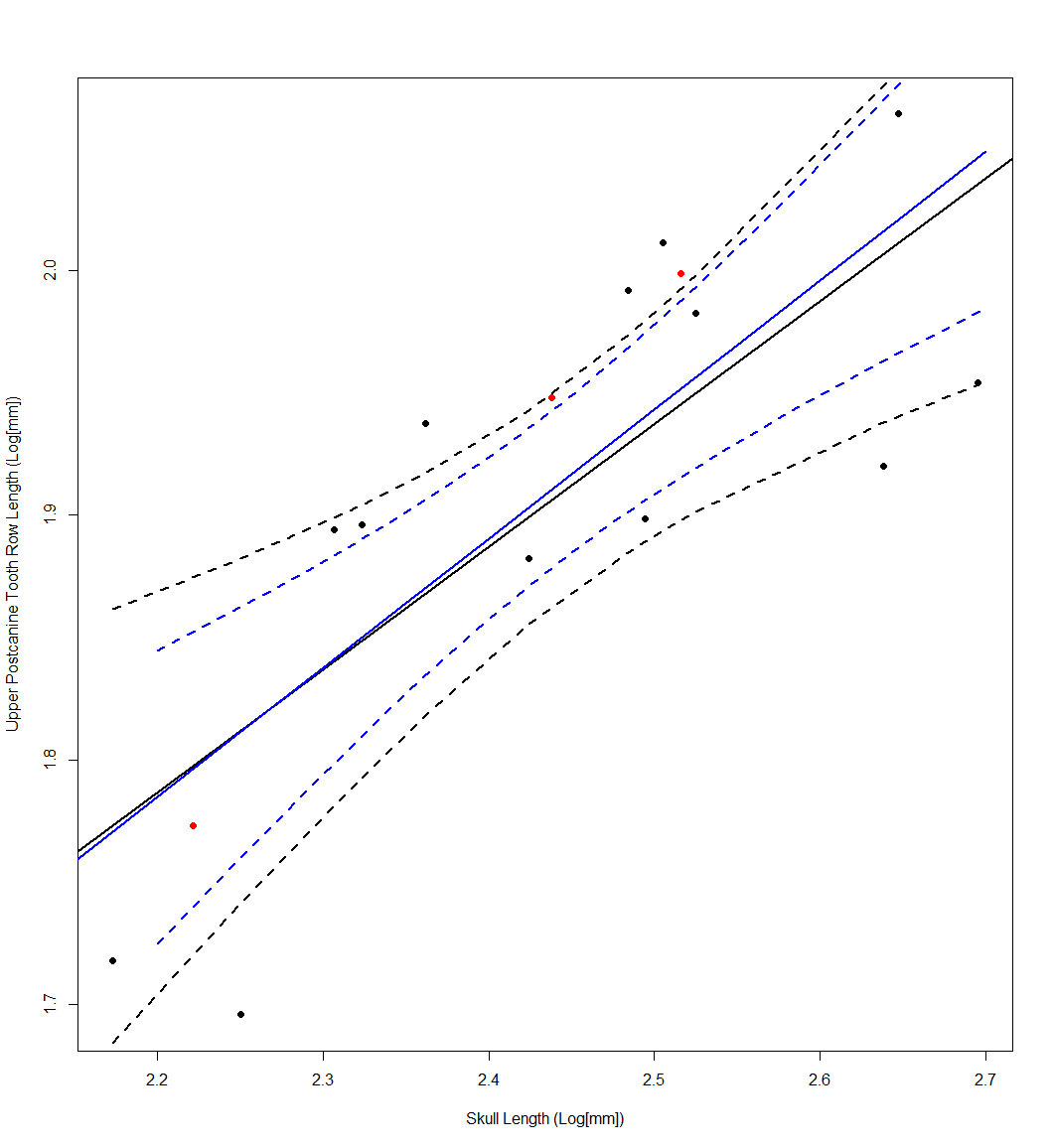


FIGURE S11: Relationship between basal skull length and upper postcanine tooth row length. Distorted measurements are represented as red dots, and undistorted measurements are represented as black dots. Ordinary least squares regression of only the black points is shown in black, and the GLMM of all samples is shown in blue. 95% confidence intervals are reported for both OLS and GLMM as dotted lines of corresponding color. α_GLMM_: 0.5386^**^; and α_OLS_: 0.5026^**^. Marginally significant models are marked via ^*^, and significance is marked via ^**^.


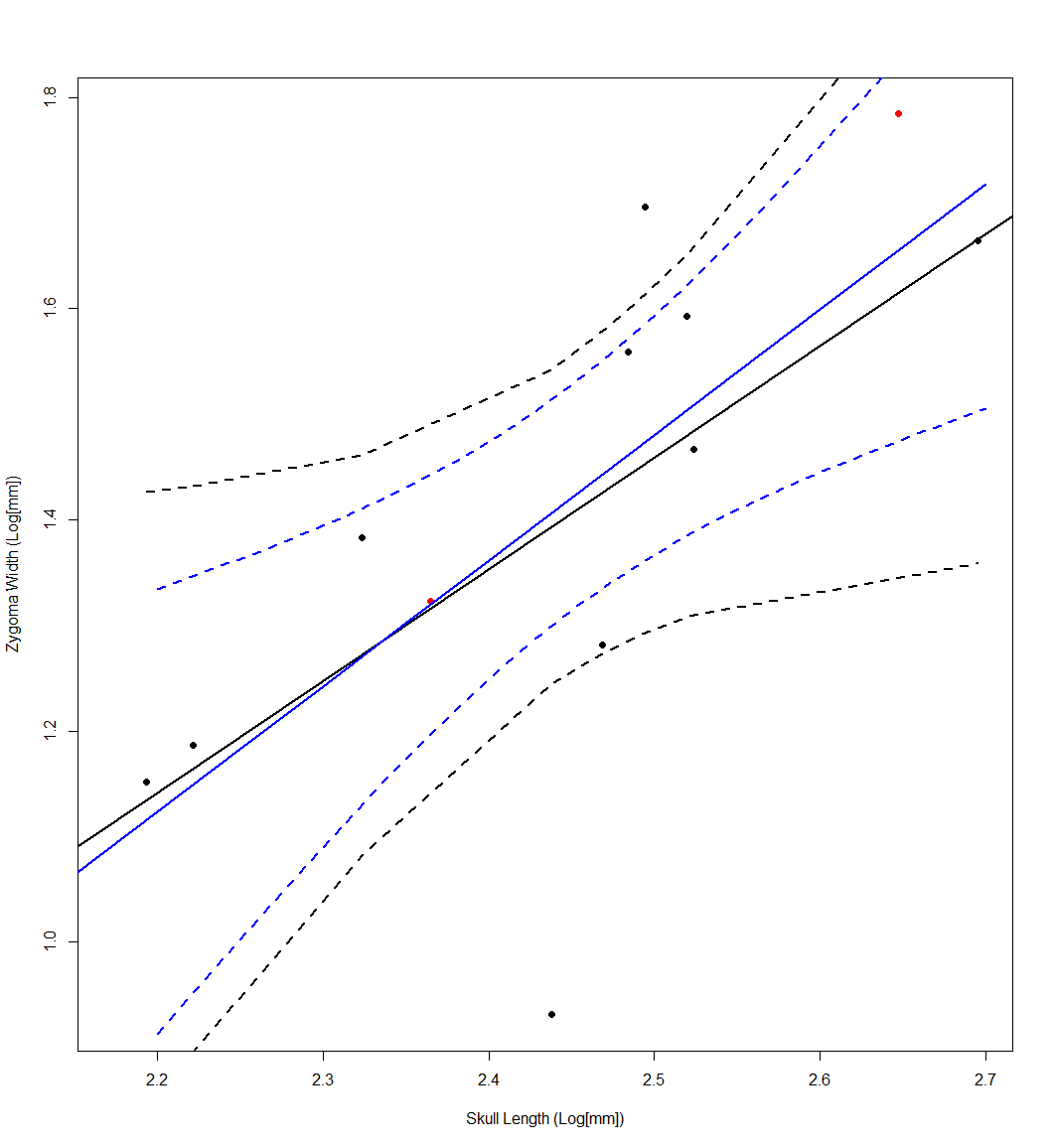


FIGURE S12: Relationship between basal skull length and zygoma width. Distorted measurements are represented as red dots, and undistorted measurements are represented as black dots. Ordinary least squares regression of only the black points is shown in black, and the GLMM of all samples is shown in blue. 95% confidence intervals are reported for both OLS and GLMM as dotted lines of corresponding color. α_GLMM_: 1.1894; and α_OLS_: 1.059. Marginally significant models are marked via ^*^, and significance is marked via ^**^.


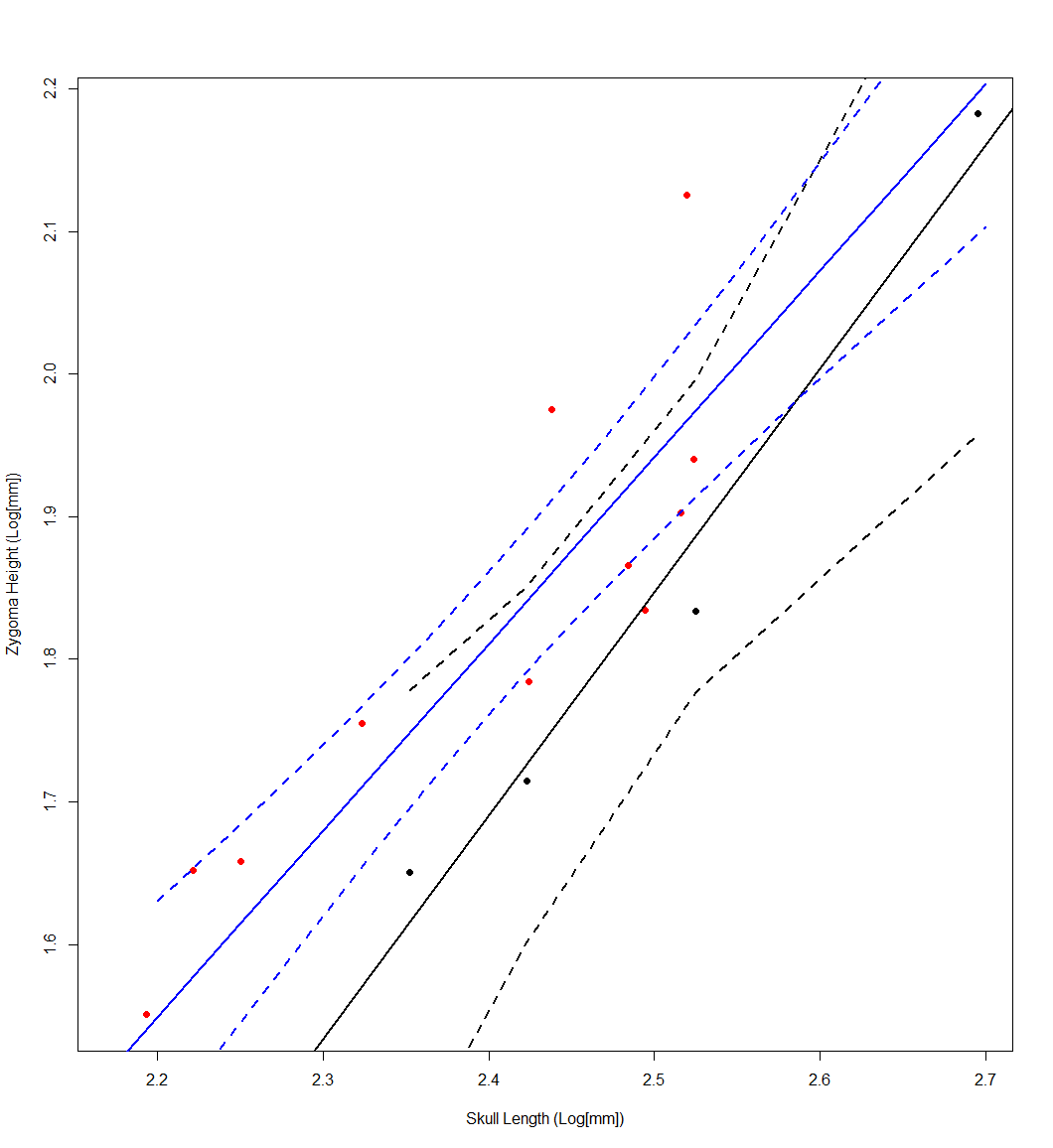


FIGURE S13: Relationship between basal skull length and zygoma height. Distorted measurements are represented as red dots, and undistorted measurements are represented as black dots. Ordinary least squares regression of only the black points is shown in black, and the GLMM of all samples is shown in blue. 95% confidence intervals are reported for both OLS and GLMM as dotted lines of corresponding color. α_GLMM_: 1.3084^*^; and α_OLS_: 1.5675^*^. Marginally significant models are marked via ^*^, and significance is marked via ^**^.

**Dental microwear**

We include additional images from the labial side of an isolated postcanine, attributed to MCZ VPRA-4470. Below images are from four separate casts made from two different molds. Each of the molds captures the same surface and orientation of the tooth, and the multiple molds/casts were made to ensure that textures are reflective of the actual dental morphology, and not an artifact of the molding and casting process.
